# From the Birkeland–Eyde process towards energy-efficient plasma-based NO_*X*_ synthesis: a techno-economic analysis

**DOI:** 10.1039/d0ee03763j

**Published:** 2021-03-31

**Authors:** Kevin H. R. Rouwenhorst, Fatme Jardali, Annemie Bogaerts, Leon Lefferts

**Affiliations:** Catalytic Processes & Materials, MESA+ Institute for Nanotechnology, University of Twente P.O. Box 217 7500 AE Enschede The Netherlands k.h.r.rouwenhorst@utwente.nl l.lefferts@utwente.nl; Research Group PLASMANT, Department of Chemistry, University of Antwerp Universiteitsplein 1 B-2610 Wilrijk-Antwerp Belgium fatme.jardali@uantwerpen.be annemie.bogaerts@uantwerpen.be

## Abstract

Plasma-based NO_*X*_ synthesis *via* the Birkeland–Eyde process was one of the first industrial nitrogen fixation methods. However, this technology never played a dominant role for nitrogen fixation, due to the invention of the Haber–Bosch process. Recently, nitrogen fixation by plasma technology has gained significant interest again, due to the emergence of low cost, renewable electricity. We first present a short historical background of plasma-based NO_*X*_ synthesis. Thereafter, we discuss the reported performance for plasma-based NO_*X*_ synthesis in various types of plasma reactors, along with the current understanding regarding the reaction mechanisms in the plasma phase, as well as on a catalytic surface. Finally, we benchmark the plasma-based NO_*X*_ synthesis process with the electrolysis-based Haber–Bosch process combined with the Ostwald process, in terms of the investment cost and energy consumption. This analysis shows that the energy consumption for NO_*X*_ synthesis with plasma technology is almost competitive with the commercial process with its current best value of 2.4 MJ mol N^−1^, which is required to decrease further to about 0.7 MJ mol N^−1^ in order to become fully competitive. This may be accomplished through further plasma reactor optimization and effective plasma–catalyst coupling.

## Abstract 2

Plasma-based NO_*X*_ synthesis can be combined with renewable electricity for decarbonized fertilizer production. Renewed research interest makes it increasingly competitive with the conventional process.

## Graphical abstract

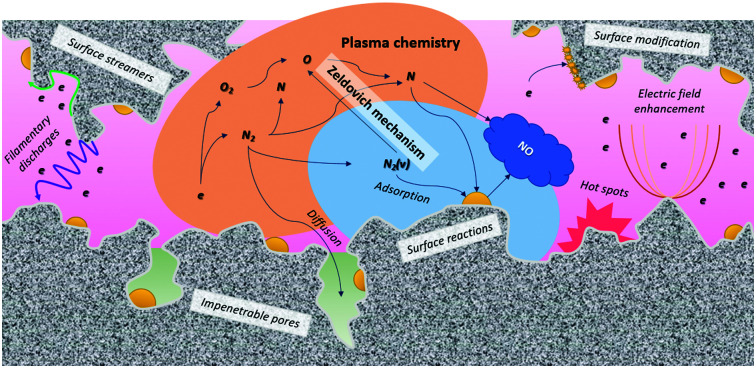

**Broader context**

Industrial nitrogen fixation was first commercialized as the plasma-based Birkeland–Eyde process about a century ago, although this process was eventually outcompeted by the Haber–Bosch process due to the lower energy consumption for nitrogen fixation of the Haber–Bosch process. Nitrogen fixation is currently highly centralized, due to the high temperature and high pressure synthesis of ammonia *via* the Haber–Bosch process. Due to the emergence of low cost renewable electricity from solar and wind, there is renewed interest in decentralized opportunities for electricity-driven nitrogen fixation. In recent years, computational studies have greatly enhanced the understanding of plasma-based nitrogen fixation. This has allowed for optimized plasma reactors with reduced energy consumption for plasma-based NO_*X*_ synthesis. This has spurred renewed interest in the plasma-based nitrogen fixation process for decentralized and on-demand fertilizer production. The recent developments are discussed in the current analysis paper, as well as energy consumption targets for renewed commercialization of plasma-based nitrogen fixation.

## Introduction

**Fig. 1:**
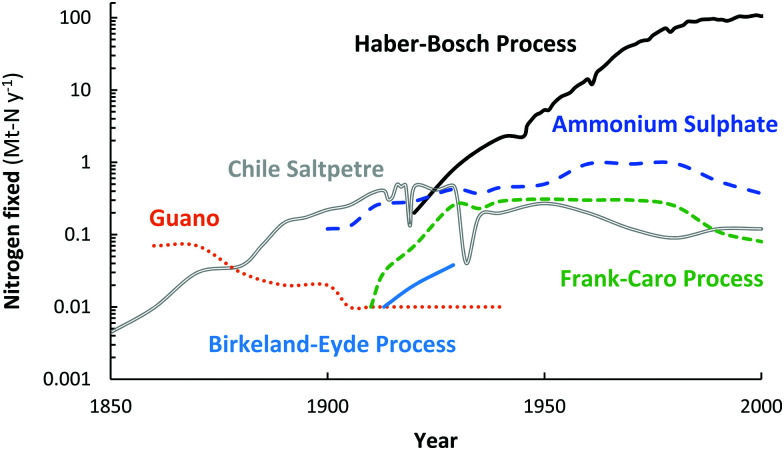
Annual consumption of fixed nitrogen from various natural sources and from industrial nitrogen fixation technologies. Original sources.^2,4,5^

For over a century, nitrogen (N_2_) has been industrially fixed into reactive nitrogen (N_r_) compounds to increase agricultural yields.^1^ In order to artificially fix atmospheric N_2_, different attempts have been made throughout the years, including the Birkeland–Eyde (B–E) process that produces NO_*X*_,^2^ the Frank–Caro (F–C) process that produces calcium cyanamide,^3^ and the Haber–Bosch (H–B) process that produces ammonia (NH_3_),^4^ among others. Nowadays, nitrogen is almost exclusively fixed *via* the Haber–Bosch process.^4^ An overview of the annual consumption of fixed nitrogen from various natural sources and from industrial nitrogen fixation technologies is shown in Fig. 1. Guano and Chile saltpetre are natural sources of fixed nitrogen, mostly derived from Chile and Peru.^4^ Ammonium sulphate is a by-product of coke ovens and of caprolactam production.

In 1903, the first synthetic plasma-based NO_*X*_ synthesis process was developed and tested in Christiania University (nowadays known as the University of Oslo) by Kristian Birkeland and Samuel Eyde. In the B–E process, air was passed through an electric arc, *i.e.*, a thermal plasma, thereby producing nitrogen oxide (NO) and nitrogen dioxide (NO_2_) (eqn (1) and (2)). Thereafter, NO_2_ was concentrated and absorbed in water to form nitric acid (HNO_3_) (eqn (3)).

Nitric acid can also be produced *via* the combined Haber–Bosch (H–B) and Ostwald process. In the H–B process, ammonia (NH_3_) is synthesized from hydrogen (H_2_) and atmospheric nitrogen (N_2_) (eqn (4)). The NH_3_ produced by the H–B process is then oxidized in the Ostwald process to form NO and NO_2_ (eqn (2) and (5)). Subsequently, the NO_2_ is absorbed in water to from HNO_3_. In both processes, the resulting product is HNO_3_, which can be neutralized with NH_3_ to form ammonium nitrate (NH_4_NO_3_) (eqn (6)). NH_4_NO_3_ is primarily used as a fertilizer for agricultural activity and as an explosive for the mining industry. NH_4_NO_3_ production accounts for about 75–80% of the HNO_3_ produced.^6^ Further uses of HNO_3_ include nitration reactions, its usage as oxidant and as rocket propellant.

1 N_2_+ O_2_ ⇌ 2NO. with Δ*H*^o^_r_ = 180 kJ mol^−1^.

2 2NO + O_2_ → 2NO_2_. with Δ*H*^o^_r_ = −114 kJ mol^−1^.

3 3NO_2_ + H_2_O → 2HNO_3_ + NO. with Δ*H*^o^_r_ = −117 kJ mol^−1^.

4 3H_2_ + N_2_ → 2NH_3_. with Δ*H*^o^_r_ = −92 kJ mol^−1^.

5 4NH_3_ + 5O_2_ → 4NO + 6H_2_O. with Δ*H*^o^_r_ = −905 kJ mol^−1^.

6 NH_3_ + HNO_3_ → NH_4_NO_3_.

Throughout the years, different factors played a role in the abandonment of the plasma-based B–E process in favour of the fossil-fuel powered H–B technology, including (i) emergence of low-cost fossil fuels such as coal and natural gas, (ii) the substantially lower energy cost for nitrogen fixation *via* the thermochemical H–B process (about 0.5–0.6 MJ mol N^−1^) as compared to the plasma-based B–E process (about 2.4–3.1 MJ mol N^−1^),^7–10^ (iii) the higher capital investment for the B–E compared to the combined H–B and Ostwald process,^2^ and (iv) the higher maintenance cost of the B–E reactor.^2,11^ Therefore, NO_*X*_ production *via* NH_3_ produced in the H–B process is more cost effective despite the fact that this is actually a detour. Nitrogen in N_2_ (oxidation state 0) is first reduced to ammonia (oxidation state −3), where after it is oxidized again to NO (oxidation state +2); in fact H_2_ is burnt in this sequence to drive the overall reaction. Instead, a direct route from N_2_ (oxidation state 0) to NO (oxidation state +2) in eqn (1) would be an elegant shortcut, which has the potential to be more efficient.

The H–B technology substantially increased the agricultural productivity and it succeeds in sustaining about 50% of the world population.^12^ Nevertheless, the H–B process suffers from its poor scalability for decentralized production. Thus, industrial plants typically produce at least 100 t-NH_3_ per day.^5^ Furthermore, the H–B process operates at high temperatures and high pressures (350–500 °C and 100–300 bar), implying operation with varying load from intermittent renewables is difficult. Therefore, current research focuses on enabling load variation,^13^ and on NH_3_ synthesis under milder conditions.^14^ Eventually, the H–B process may be replaced by a single-pass thermo-catalytic NH_3_ synthesis process or electrochemical NH_3_ synthesis.^15,16^

The emergence of low cost and intermittent renewable electricity may change the preferred choice of technology. Plasma technology offers potential benefits, such as fast turning on and off, and scalability for small communities.^9,17^ The aim of this paper is to evaluate whether plasma-activated NO_*X*_ synthesis can become a feasible alternative for nitrogen fixation again in the 21st century, just like it was at the start of the 20th century. We identify how the state-of-the-art plasma nitrogen fixation process compares to the benchmark thermo-catalytic H–B process with the subsequent thermochemical Ostwald process. For this purpose, we will first explain the principles and state-of-the-art of the B–E process, the H–B process and the Ostwald process.

### The Birkeland–Eyde process

**Fig. 2:**
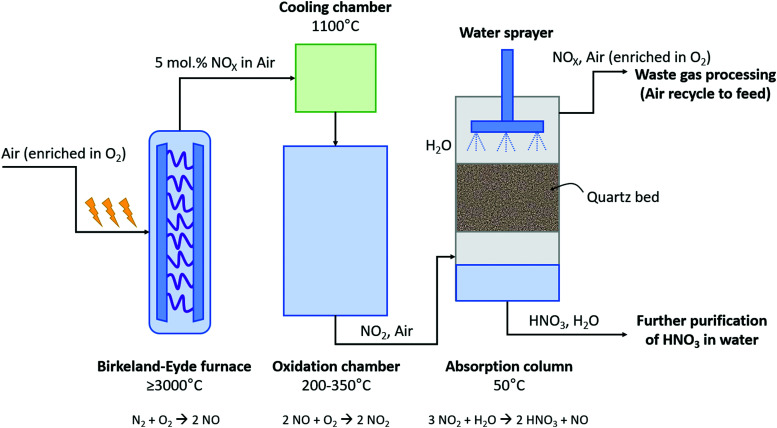
Process scheme for the Birkeland–Eyde industrial nitrogen-fixation process. Inspired by Patil *et al.*^9^

The B–E process was the first nitrogen fixation process to operate commercially with hydropower in Niagara Falls (Canada). The power supplied to the B–E plant increased from 2.24 kW in 1903 to 238.6 MW in 1928. This commercial plant succeeded in fixing 38 kt-N year^−1^^2^. About 175 t-air was required to fix 1 t-N *via* the B–E process.^9^ The B–E process consumed about 2.4–3.1 MJ mol N^−1^ and produced 1–2 mol% NO.^9,17^ A process scheme for the B–E process is shown in Fig. 2. Air was converted to NO in an electric arc formed between two co-axial, water-cooled copper electrodes placed between the poles of a strong electromagnet inside a furnace, for which various alternative configurations were considered.^9^ Rapid quenching of the dilute nitrogen oxides to 800 – 1000 °C was applied at the reactor outlet to prevent reverse reactions (*i.e.*, converting NO back to N_2_ and O_2_).^2^ The heat of the reaction was recovered in waste heat boilers. Afterwards, oxidation of NO to NO_2_ took place at a slow rate in a large oxidation chamber. Since the absorption capacity increases with decreasing temperature, the mixture of NO and NO_2_ leaving the economizer at about 200 °C was further cooled to 50 °C in cooling towers before entering the absorption towers. Finally, NO_2_ gas was absorbed in water to produce a solution of HNO_3_. The final stream contained about 30% HNO_3_ in water.^2^ The unabsorbed NO_*X*_ was passed through alkaline absorption columns for further absorption. Despite this second absorption step, about 3% of the produced NO_*X*_ was purged to the atmosphere.

Many ideas have been suggested to reduce the energy consumption of NO_*X*_ production and improve the performance of the B–E process, as for example the use of a 50–50% mixture of N_2_ and O_2_, preheating the inlet gas, applying heat recovery from process gas and operating the furnace at elevated pressures.^9^ However, not only the plasma reactor is a major contributor to the investment and the energy cost of the B–E process, as also the absorption towers, especially the acid absorption towers, contribute significantly to the CapEx and OpEx.^18^ According to a 1922 report on nitrogen fixation, the absorption columns compose over 40% of the CapEx and about 30% of the OpEx.^18^ These absorbers were costly due to the low concentration of NO_*X*_ at the outlet of the plasma reactor. However, this technology has been optimized for the Ostwald process in previous decades, which could be used in combination with the B–E process as well. More recently, adsorbents, such as BaO, have been used to concentrate NO_*X*_ for car exhaust catalysts.^19^ Through temperature swing adsorption (TSA) or pressure swing adsorption (PSA), the concentration of NO_*X*_ can be increased by using such solid sorbents. Possibly, such solid sorbents can replace or minimize the use of the costly absorption columns in the B–E process.

### The Haber–Bosch process combined with the Ostwald process

In 1908, Haber and Le Rossignol demonstrated the feasibility of direct synthesis of 2 kg-NH_3_ day^−1^ from N_2_ and H_2_ with a table top system operating at 500–550 °C and 100–200 atm, in the presence of an osmium catalyst.^1^ In the following years, Mittasch and co-workers developed the multicomponent iron catalyst, a less poisonous and more abundant material, as an alternative to osmium for NH_3_ synthesis,^20,21^ while Bosch and co-workers solved engineering challenges regarding the operation with H_2_ at high pressures.^22^ In 1913, the first ammonia synthesis plant started operating according to the H–B process at BASF in Oppau, Ludwigshafen.^20^ Nowadays, the H–B process starting from methane consumes about 0.5–0.6 MJ mol N^−1^. This is the total energy content of the feed methane, of which about two third is transformed into hydrogen, while the remainder is used for heating during the steam methane reforming section for H_2_ production, as discussed below.^23^ The energy content of the ammonia product is only 0.32 MJ mol N^−1^, implying significant heat generation during ammonia synthesis from methane. On the other hand, the H–B process starting from H_2_O and N_2_ also consumes about 0.5–0.6 MJ mol N^−1^ nowadays. The theoretical minimum energy consumption for NH_3_ synthesis from H_2_O and N_2_ is 0.35 MJ mol N^−1^. The overall yield of the H–B process is typically 97–99%, depending on the source of H_2_ used.^15^

**Fig. 3:**
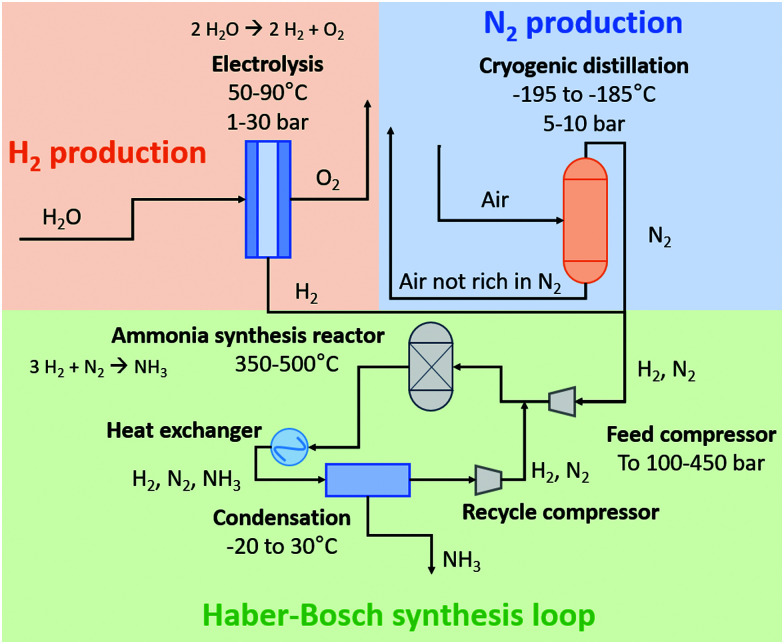
Schematic diagram of the electrolysis-based Haber–Bosch process. For details, see text. Inspired by ref. 15.

Schematic diagrams for a natural gas-based H–B process and an electrolysis-based H–B process are shown in Fig. 3. In the former method, H_2_ is produced from methane (CH_4_) *via* steam methane reforming (SMR), in which a mixture of CO, CO_2_, and H_2_ is produced. Typically, CH_4_ is first converted with H_2_O to CO and H_2_ in a tubular reformer at 850–900 °C and 25–35 bar, after which the last portion of CH_4_ conversion is performed by partial oxidation with air at 900–1000 °C, thereby introducing N_2_ in the gas mixture. The CO is then converted with H_2_O to CO_2_ and H_2_ in a two-stage water–gas-shift reactor, after which CO_2_ is removed. Traces of CO are converted to CH_4_ in a methanation step just before the synthesis loop, preventing deactivation of the ammonia synthesis catalyst. The feed gas, mainly composed of H_2_ and N_2_, is then compressed and fed to the ammonia synthesis loop operating at typically 100–300 bar, in which the reactants are fed to the ammonia synthesis reactor with iron-based catalysts operating at 350–500 °C. About 15–20% of the feed gas is converted to NH_3_. The reactor effluent is then cooled down to ambient temperature to condense the NH_3_ out. The remaining gas is recycled to the NH_3_ synthesis reactor. This process scheme of NH_3_ synthesis would be similar to the electrically-driven system. Here, H_2_ is produced by electrolysis. Purified N_2_ in the electrolysis-based process is produced in a separate unit by pressure swing adsorption (PSA) or cryogenic distillation.^14,24^ Due to the different feedstocks for the SMR-based Haber–Bosch process and the electrolysis-based Haber–Bosch process, the heat integration between the process components changes substantially.

**Fig. 4:**
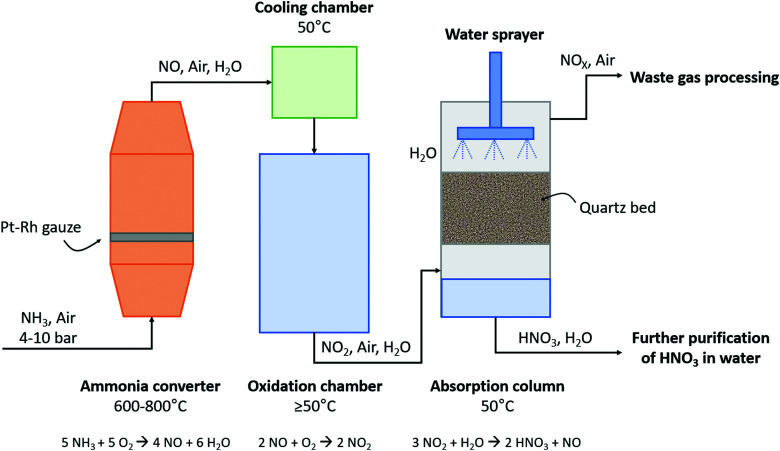
Schematic diagram of the Ostwald process. For details, see text. Reproduced from ref. 25.

The subsequent oxidation process was developed by Wilhelm Ostwald, who patented the ammonia oxidation process in 1902.^6^ In this process, ammonia is oxidized in the presence of a rhodium–platinum gauze to form NO and H_2_O at 600–800 °C and 4–10 atm. Afterwards, NO is cooled to about 50 °C and subsequently oxidized to NO_2_ and absorbed in H_2_O, producing dilute HNO_3_. The untreated NO is recycled, while the HNO_3_ is concentrated by distillation. The overall yield of the Ostwald process is typically 98%. A process scheme of the Ostwald process is shown in Fig. 4, which is similar to the B–E process (see Fig. 2), although less absorption steps are required due to the higher NO_2_ concentration after the oxidation reactor.

### State-of-the-art of plasma-based NO_*X*_ synthesis

As discussed above, plasma-based NO_*X*_ synthesis was commercialized by Birkeland and Eyde in 1903,^26,27^ and the energy consumed by the electric arc to generate a thermal plasma for NO synthesis is 2.4–3.1 MJ mol N^−1^^7–9^. Hereafter we will discuss the state-of-the-art of plasma-based NO_*X*_ synthesis, as well as potential avenues for improvements.

### Plasma types and comparison of energy consumption

Various plasma types can be distinguished, namely thermal plasmas, warm plasmas, and non-thermal plasmas. In a thermal plasma, the electrons and the heavier plasma species (molecules, radicals, and ions) are in thermal equilibrium, forming a quasi-neutral plasma bulk. The temperature in a thermal plasma is typically high (order of 10^4^ K). The highest NO equilibrium concentration (about 5 mol%) can be achieved at a gas temperature near 3500 K and at 1 atm.^8^ The NO formed is also prone to decomposition after the plasma, forming N_2_ and O_2_ again. Therefore, rapid quenching of the gas is required at a rate of several millions of Kelvins per second.^28,29^ However, even if thermal plasma reactors are optimized, the theoretical minimum energy consumption for thermal plasmas is 0.72 MJ mol N^−1^, which means that the energy efficiency of thermal plasma cannot compete with nitric acid produced from an electrolysis-based Haber–Bosch process (about 0.6 MJ mol N^−1^). Here, the energy consumption refers to the electricity input for nitrogen fixation. The theoretical minimum energy consumption for thermal plasmas is based on the assumption that both N_2_ and O_2_ dissociate completely in the plasma, considering the bond-dissociation energies of N_2_ (945 kJ mol^−1^) and O_2_ (498 kJ mol^−1^).

In a non-thermal plasma, on the other hand, the electrons are not in equilibrium with the heavier plasma species, resulting in a substantially higher electron temperature as compared to the gas temperature, which is typically near room temperature. This potentially allows for selectively activating molecules with a strong chemical bond, such as N_2_ (about 9.79 eV).^30^ This is relevant for NO formation, as breaking the triple N≡N bond is rate-limiting for the formation of NO. The O_2_ dissociation step takes place more easily, because of the somewhat weaker O=O double bond (about 5.12 eV). Depending on the actual electron temperature, electrons can excite the molecules to various vibrational and electronic states. In typical non-thermal plasmas, such as dielectric barrier discharges (DBDs), the electron temperature is typically several eV, which mainly gives rise to electronic excitation.^17^

In between thermal and non-thermal plasmas, we can identify so-called warm plasmas, such as gliding arc (GA) and microwave (MW) plasmas, in which the electron temperature is still higher than the gas temperature, but the latter can be several 1000 K.^17^ The electron temperature is typically 1–2 eV,^17^ which is more beneficial for vibrational excitation of the molecules than in non-thermal plasmas (see eqn (7) for vibrational excitation). This gives rise to more efficient NO_*X*_ formation in warm plasmas.

Indeed, the NO formation rate *via* the reaction of atomic oxygen with N_2_ by the so-called vibrationally-promoted Zeldovich mechanisms (eqn (8)) is enhanced upon increasing the population of N_2_ vibrational levels in the plasma. The chain mechanism of NO synthesis is closed by the exergonic reaction given by Equation 9.^28,31^ The sum of eqn (8) and (9) then gives a net energy consumption of 0.2 MJ mol N^−1^ for NO_*X*_ synthesis (*cf.*Table 1), *i.e.*, lower than NO_*X*_ synthesis *via* the electrolysis-based Haber–Bosch process combined with the Ostwald process. Therefore, exploiting the non-equilibrium phenomena in a plasma is a promising approach to increase the energy efficiency of plasma-based processes for nitrogen fixation.

**Table 1:** Comparison of energy consumption for various production methods for nitric acid (best available technology, and minimum energy consumption). The best available technology refers to industrial practice and laboratory results. * See the ESI

Technology	Best available technology (MJ mol N^−1^)	Minimum energy consumption* (MJ mol N^-1^)
Thermochemical process (electrolysis-based Haber–Bosch + Ostwald)	0.6^15^	0.35^32^
Thermal plasma (Birkeland–Eyde process)	2.4–3.1^7–9^	0.72
Warm plasma (Gliding arc reactor)	2.4^33^	0.5^34^
Plasma with only vibrationally-promoted Zeldovich mechanism (only vibrational excitations in N_2_)	—	0.2^8^

It should also be noted that unproductive electronic excitation and ionization channels in real plasma reactors lead to a higher minimum energy consumption than for an hypothetical plasma reactor operating exclusively *via* the vibrationally-promoted Zeldovich mechanism (eqn (8)). The distribution of productive and unproductive N_2_ activation channels leads to a theoretical minimum energy consumption of about 0.5 MJ mol N^−1^ (see Table 1) for a gliding arc plasma reactor, which is a warm plasma type.^28,33,34^ The different plasma activation channels for N_2_ and O_2_ in various plasma reactors are shown in Fig. 6.

In practice, the energy consumption is even higher, which is due to vibrational–translational relaxation (hence depopulating the N_2_ vibrational levels), and NO_*X*_ decomposition after the plasma if the temperature does not drop fast enough. Plasma radicals may also recombine to form O_2_ and N_2_ again, implying all energy is lost as heat. Lastly, decomposition of NO_*X*_ products in the plasma will further limit the energy efficiency. With increasing NO_*X*_ concentration, the probability of plasma-activation of NO_*X*_ increases, thereby promoting the reaction back to N_2_ and O_2_.

7 e^−^ + N_2_ → e^−^ + N_2_(v).

8 O + N_2_(v) → NO + N.

with *E*_a_ ≈ Δ*H*_r_ ≤ 3 eV per molecule (note: 3 eV is the barrier for a ground-state N_2_ molecule, and the barrier decreases upon increasing vibrational excitation of N_2_)

9 N + O_2_ → NO + O.

with *E*_a_ ≈ 0.3 eV per molecule and Δ*H*_r_ ≈ −1 eV per molecule^28^

The enthalpy of formation for NO is 90 kJ mol N^−1^ and any addition of energy input above that level leads to the formation of heat. Thus, even in case of the Zeldovich mechanism with an energy consumption of 0.2 MJ mol N^−1^, 55% of the energy in the reactor is lost as heat. In case of thermal dissociation of the triple N≡N bond (945 kJ mol^−1^) and double O=O bond (498 kJ mol^−1^), only 12% of the energy is stored in the N=O bond whereas 88% in converted to heat.

### Plasma catalysis

A potential avenue to improve the energy efficiency of the process beyond optimizing the plasma is the introduction of a catalyst. Catalysts are used in most chemical processes to decrease the reactor size, as well as to operate at milder operating conditions and to lower t energy requirement. Various authors have attempted the use of metal and metal oxide catalysts for plasma-based NO_*X*_ synthesis.^35,36^ However, up till now, results are inconclusive on whether there is an actual catalytic effect rather than a change in the physiochemical plasma properties d to the introduction of a packing material into the reactor.^8,36,37^ A change of packing material is known to modify the plasma properties, and thereby the conversion.^38^ Some synergistic effects between plasma and catalyst have however been proposed. Rapakoulias *et al.*^35^ investigated NO synthesis in the presence of transition metal oxides, such as molybdenum trioxide (MoO_3_) and tungsten trioxide (WO_3_) catalysts (*e.g.* n-type semiconductors). The authors proposed that the vibrationally excited N_2_ molecules undergo dissociative adsorption on the catalytic surface (eqn (10)). This may occur because n-type semiconductors donate electrons because of their easy ionization. Therefore, the adsorbed molecule can accept electrons to its anti-bonding π* orbital, leading to its pre-dissociation.^39^ Then, the atomic nitrogen may react with surface oxygen, forming NO upon desorption (eqn (11)). The oxygen vacancy can then be replenished by oxygen from the gas phase (eqn (12)), thereby oxidizing the transition metal surface, according to a Mars–van Krevelen redox mechanism.^40^10
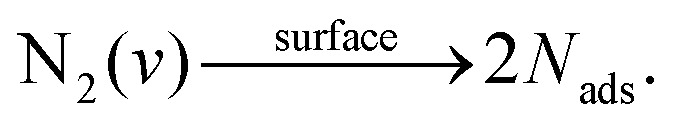
11
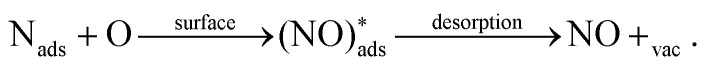
12
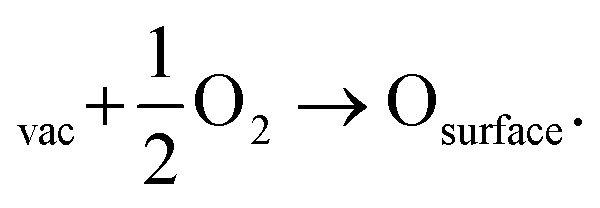
It should be noted, however, that the dissociative sticking probability of N_2_ is probably very low on oxide surfaces, even upon substantial activation of N_2_*via* vibrational or electronic excitation. The dissociative sticking probability on Ru(0001), a metal that has thermal activity for N_2_ dissociation, for N_2_ pre-activated with 300–400 kJ mol^−1^ is as low as 10^−3^–10^−2^^41, 42^. For W(110), a metal that is much less noble, the dissociative sticking probability is only 0.35 upon pre-activation of 100 kJ mol^−1^^43^. As oxides are much less able to dissociate N_2_ compared to metals, the sticking probability of N_2_ on oxides is even much lower, so most of the collisions of activated N_2_ molecules with the oxide surface will lead to energy relaxation instead of N_2_ dissociation. This will be a major pathway for energy loss.^44^

A limitation of a thermally-active catalyst is that it always catalyses not only the NO_*X*_ synthesis reaction but also the reverse decomposition reaction.^45^ As the equilibrium at mild conditions is completely towards the formation of N_2_ and O_2_, a metal catalyst with thermal catalytic activity will in principle mainly form N_2_ and O_2_ under mild conditions.^46^ The presence of a surface could improve the performance only if it would enhance an irreversible reaction step, *e.g.* a quenching reaction of a highly activated species, leading to the formation of NO_*X*_.^47^ This can potentially be achieved with metal oxide catalysts, or metals inactive for NO_*X*_ decomposition such as Ag and Au. However, at ambient temperatures, a catalytic effect was not observed for NO_*X*_ synthesis on alumina-supported W-, Co- and Pb-oxides in a dielectric barrier discharge (DBD) reactor,^36^ and any change in activity must be attributed to modifications in the physiochemical plasma properties due to the introduction of a packing material into the reactor. On the other hand, metal oxides become active for NO decomposition at elevated temperatures.^45,48–50^

### Performance of various plasma reactors

Various plasma types and plasma reactors have been investigated for NO_*X*_ production after the earlier research on thermal plasma (*i.e.*, the electric arc).^26,27,33,51^ This includes spark discharges,^52–55^ radio-frequency crossed discharge,^56^ laser-produced discharge,^57^ corona discharges,^52,58^ glow discharges,^53,59^ (packed bed) dielectric barrier discharges (PB) DBD,^36,53^ (pulsed) (gliding) arc discharges,^34,53,60–62^ microwave (MW) discharges,^63–65^ and plasma jets in contact with water.^66–76^

**Fig. 5:**
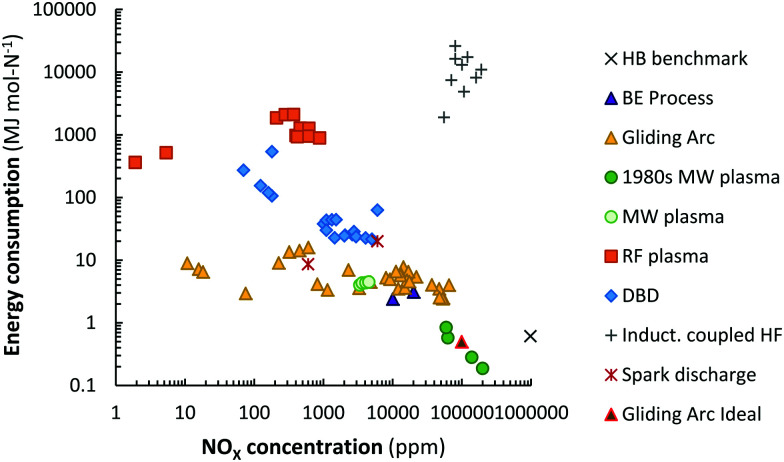
Comparison of energy consumption for NO production in various plasma reactors. Original references: 1980s low pressure MW plasma,^64,65,77,78^ MW plasma,^63^ gliding arc,^33,34,53,60,62,79–82^ RF plasma,^83,84^ DBD,^36,53,85,86^ inductively coupled HF,^35^ spark discharge.^54,55^

A summary of the reported energy consumption and the product concentration in various plasma reactors is listed in Table 2. Additionally, the reported NO_*X*_ concentration and energy consumption are shown in Fig. 5. A distinction is made between various types of plasma reactors.

**Table 2:** Comparison of energy consumption for NO production in various plasma reactors

Plasma type	Product (concentration)	Energy cost (MJ mol N^−1^)	Ref.
Electric arc (Birkeland–Eyde)	NO (2%)	2.4–3.1	26,27,51
Spark discharge	NO and NO_2_	20.27, 40	52,55
Transient spark discharge	NO and NO_2_	8.6	54
Pin-to-plane ns-pulsed spark discharge	NO and NO_2_	5.0–7.7	53
Radio-frequency crossed discharge	HNO_3_	24–108	56
Laser-produced discharge	NO and NO_2_	8.9	57
(Positive/negative) DC corona discharge	NO and NO_2_	1057/1673	52
Pulsed corona discharge	HNO_3_	186	58
Pin-to-plane DC glow discharge	NO and NO_2_	7	53
Pin-to-pin DC glow discharge	NO and NO_2_ (0.7%)	2.8	59
Dielectric barrier discharge	NO and NO_2_ (0.6%)	56–140	53
Packed dielectric barrier discharge	NO and NO_2_ (0.5%)	18	36
DC plasma arc jet	NO (6.5%)	3.6	60
Propeller arc	NO and NO_2_ (0.4%)	4.2	53
Pulsed milli-scale gliding arc	NO and NO_2_ (1–2%)	2.8–4.8	61,62
Gliding arc plasmatron	NO and NO_2_ (1.5%)	3.6	34
Rotating gliding arc	NO and NO_2_ (5.4%)	2.5	33
Microwave plasma	NO and NO_2_ (0.6%)	3.76	63
Microwave plasma with catalyst	NO (6%)	0.84	64
Microwave plasma with magnetic field	NO (14%)	0.28	65

Among the different plasma types, warm plasmas, such as gliding arcs (GA), atmospheric pressure glow discharges (APGD) and microwave plasmas (MW), have been explored extensively for gas conversion applications.^17^ As explained above, warm plasmas are a special type of plasma that include both thermal and non-thermal plasma characteristics. The gas temperature is typically a few 1000 K, while the electron temperature is still higher (1–2 eV), thus, providing warm plasmas with non-equilibrium (or non-thermal) characteristics. However, the vibrational temperature is (nearly) equal to the gas temperature, resulting in vibrational–translational (VT) equilibrium.^87,88^ Therefore, warm plasmas are also known as quasi-thermal plasmas.

**Fig. 6:**
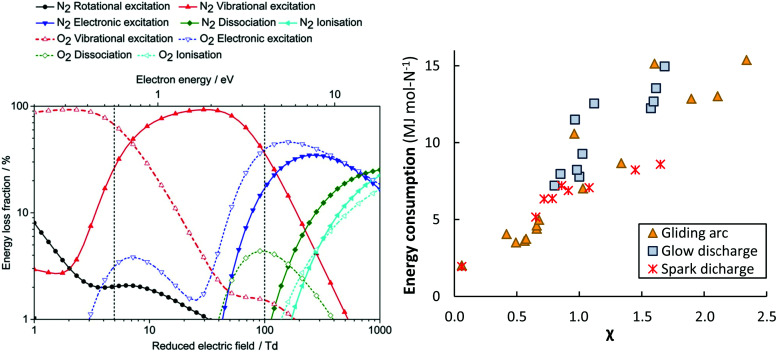
Left: The dominant plasma-activation channels in 50 : 50 N_2_ : O_2_ stream. Reproduced from ref. 62. Reduced electric fields of 5–100 *T*_d_ correspond to GA and MW plasmas, while the region above 100 *T*_d_ corresponds to a DBD reactor.^17^ Right: The apparent energy cost as function of the *χ* factor, as proposed by Pei *et al.*^53^ Original reference: gliding arc,^34,53,62,80,81,91–95^ glow discharge,^53^ spark discharge.^53,54^

Different GA reactor configurations have shown promise for gas conversion applications.^17,34,61,62,89,90^ GA plasmas are characterized by reduced electric fields below 100 Td, resulting in electron energies around 1 eV. Such electron energies are most beneficial for vibrational excitation of the gas molecules (see Fig. 6a).^17^ Wang *et al.*^62^ investigated NO_*X*_ formation mechanisms in a pulsed-power milliscale GA reactor, while Vervloessem *et al.*^34^ studied NO_*X*_ formation in a reverse-vortex flow gliding arc plasmatron (GAP). The chemical kinetics modelling results showed that the vibrationally excited N_2_ molecules can reduce the energy barrier of the non-thermal Zeldovich mechanism O + N_2_(v) → NO + N, providing an energy-efficient way for NO production.

Moreover, the high gas temperature (>3000 K) leads to significant thermal dissociation of the lower N_2_ vibrational levels, whose vibrational distribution function exhibits a Boltzmann shape. In fact, thermal reactions are quite efficient at the high temperatures reached in GA reactors. The limitation in the overall N_2_ conversion is rather the fraction of gas treated by the GA plasma. For instance, only 15% of the gas is estimated to pass through the plasma arc in the GAP and the rest of the gas by-passes through the reactor without contacting the plasma.^90,96^ Vervloessem *et al.*^34^ reported a NO_*X*_ yield of 1.5% at an energy consumption of 3.6 MJ mol N^−1^. Through reactor optimization and by preventing the transfer of vibrational energy from N_2_ to O_2_, the authors showed that the energy consumption can potentially decrease to 0.5 MJ mol N^−1^.^34^

Janda *et al.*^54^ studied NO_*X*_ production in a transient spark discharge. This type of spark discharge starts from a streamer phase, *i.e.* a non-thermal plasma, and is subsequently transformed into short spark current pulses which generate a thermal plasma. The self-pulsing feature of the discharge avoids thermalization of the plasma.^97,98^ The spark phase is characterized by a high chemical activity due to the high electron density achieved (about 10^17^ cm^−3^). The excited nitrogen molecules (N_2_*) were observed in both the streamer and the spark phases and the energy consumption for NO_*X*_ production was 8.6 MJ mol N^−1^^54^. Pavlovich *et al.*^55^ developed a spark-glow discharge reactor, where the generated plasma discharge had a spark phase (thermal plasma) and glow phase (non-thermal plasma) in one cycle. The authors were able to control the percentage of glow phase by fine-tuning the voltage waveforms. The spark phase, which had a very high electron density and energy, generated more NO, while the glow phase promoted the oxidation of NO to NO_2_. However, the energy consumption of NO_*X*_ production was as high as 40 MJ mol N^−1^. In general, such plasma types have a limited volume, resulting in a limited fraction of the N_2_ gas being exposed to the plasma, and thus a limited amount of NO_*X*_ produced.

Packed bed DBD reactors have also been studied, because of the possibility to enhance the product selectivity and the energy efficiency by combining the plasma with a catalyst. Patil *et al.*^36^ studied NO_*X*_ production in a DBD packed with different catalyst support materials (α-Al_2_O_3_, γ-Al_2_O_3_, TiO_2_, MgO, TaTiO_3_, and quartz wool). The authors obtained the best results with a γ-Al_2_O_3_ catalyst with the smallest particle size of 250–160 μm. However, the obtained energy cost was high (18 MJ mol N^−1^) and the product yield low (0.5 mol%), compared to other atmospheric pressure plasma reactors.^36^ These poor results obtained in a DBD could be explained by the high reduced electric field, *i.e.* above 100–200 *T*_d_, which creates highly energetic electrons, resulting mainly in electronic excitation, ionization, and dissociation, instead of vibrational excitation (see Fig. 6a), and thus not exploiting the most energy-efficient NO_*X*_ formation pathway through the vibrationally-induced Zeldovich mechanism.^17^

The best results in terms of product yield and energy consumption were obtained in low-pressure MW plasmas. The energy consumption obtained in a MW plasma with catalyst was stated to be 0.84 MJ mol N^−1^ for an NO concentration of 6 mol%.^64^ The highest NO concentration of 14% and lowest energy cost of 0.28 MJ mol N^−1^ were reported for a MW plasma with magnetic field (so-called electron cyclotron resonance).^65^ However, these values were reported in the 1980s and have not been reproduced in recent years. A similar situation exists for plasma-based CO_2_ splitting, where results from the 1980s could not be reproduced with similar reactors in recent years.^99^ Therefore, the reported energy yield calculations for plasma-based NO_*X*_ synthesis in a MW plasma from the 1980s should be assessed critically.

These MW plasmas operated at reduced pressures (down to 66 mbar), which indeed promote vibrational–translational non-equilibrium, and thus the vibrational-induced Zeldovich mechanisms. Hence, this partially explains their high product yields and low energy consumption. However, the low reported energy consumptions only account for the plasma power and do not include the energy consumed by both the vacuum equipment and the reactor cooling system. Therefore, the overall energy cost of NO_*X*_ production in a MW plasma would be higher. Operation of MW reactors at higher pressures is also possible, but heat losses increase due to increased collision frequency.^100^

In 2010, Kim *et al.*^63^ reported a performance of 3.76 MJ mol mol N^−1^ and 0.6% NO_*X*_, similar to that of GA reactors, but for a MW plasma at a pressure slightly below atmospheric and for an input power between 60 and 90 W and at a fixed flow rate of 6 L min^−1^ (see Fig. 5). Power pulsing in a MW reactor may suppress unfavourable vibrational–translational relaxation, hence increasing the vibrational temperature, and thus the vibrational–translational non-equilibrium, needed for (the most energy-efficient) vibration-induced dissociation of N_2_.^101^

Pei *et al.*^53^ investigated four different plasma types, *i.e.* DBD, glow, spark and arc-type, and identified a key parameter (so-called *χ* factor, eqn (13)) that appeared to correlate the energy cost of NO_*X*_ production with a range of different discharges (see Fig. 6b). The authors showed that NO_*X*_ production efficiency can mainly be controlled by the average electric field and the average gas temperature of the discharge.13
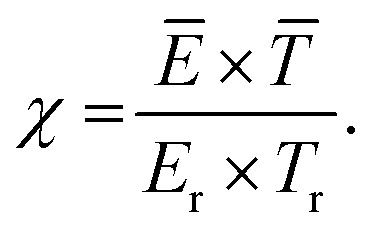
Therefore, they defined the dimensionless parameter by eqn (13), where *Ē* (kV cm^−1^) and *T̄* (K) are the average electric field and average gas temperature of the discharge under study, respectively, while *E*_r_. (*i.e.* 1.43 kV cm^−1^) and *T*_r_ (*i.e.* 1800 K) are chosen to normalize the parameter to a reference condition. The authors chose a DC glow discharge with a gap of 5 mm and a discharge current of 45 mA as a reference condition because of its simplicity and stability, *i.e.* the discharge conditions can be easily reproduced for reference. By decreasing the *χ* factor, *e.g.* by decreasing the electric field and/or the average gas temperature of the discharge, the energy cost can be reduced. The two important mechanisms that control the energy efficiency of NO_*X*_ production in any type of discharge are (i) efficient electron-impact activation of N_2_ molecules to facilitate NO_*X*_ formation, which is influenced by the electric field, and (ii) rapid thermal quenching of NO to prevent its conversion back to N_2_ and O_2_ molecules when the gas temperature drops more slowly. N atoms formed at high electric fields are an important pathway for NO_*X*_ decomposition.^62^ The authors suggested various methods to decrease the average gas temperature, such as cooling the reactor walls with water, using short duration high current pulses, and extending t discharge length.^33^

Finally, NO_*X*_ production has also been reported by plasma jets flowing in (ambient) air (or N_2_ atmosphere), and interacting with water.^66–76^ Generally, the focus of this research was on NH_3_/NH_4_^+^ formation, but NO_2_^−^ and NO_3_^−^ formation was also reported, due to the presence of oxygen. The combination of plasma jets and water potentially allows for removal of the product NO_*X*_, thereby preventing its decomposition by the plasma.

### Comparison of direct plasma-based NO_*X*_ synthesis and the Haber–Bosch process combined with the Ostwald process

In this section, we assess the techno-economic feasibility of a direct plasma-based NO_*X*_ synthesis process with subsequent conversion to HNO_3_, in comparison to an electrolysis-based Haber–Bosch process combined with the Ostwald process for HNO_3_ production. Both processes produce nitric acid from water, air, and electrical power exclusively. To the best of our knowledge, direct cost analyses comparing the direct plasma-based NO_*X*_ synthesis process and the H–B process combined with the Ostwald process have not been reported yet.^2,102^

The production capacity considered is 100 t-HNO_3_ day^−1^, *i.e.* a factor 1000 smaller than world-scale Haber–Bosch plants, at an electricity cost of 20 € MW h^−1^. The cases considered are (1) the electrolysis-based Haber–Bosch process combined with the Ostwald process (EHB + O base-case), (2) the plasma-based NO_*X*_ synthesis process at an energy cost of 2.4 MJ mol N^−1^ (PL base-case, based on the recent results of Jardali *et al.*^33^ for gliding arc plasmas), and (3) the potential plasma-based NO_*X*_ synthesis process at an energy cost of 0.5 MJ mol N^−1^ (PL potential). The energy consumption of 0.5 MJ mol N^−1^ is based on the theoretically minimum attainable energy consumption in a gliding arc reactor,^34^ as listed in Table 1.

### Capital expenditure

The capital expenditure for the electrolysis-based Haber–Bosch process and the Ostwald process (*e.g.*, the EHB + O base-case) is estimated from cost-scaling relations.^103,104^ The capital expenditure for the plasma-based NO_*X*_ synthesis process (PL) is estimated from the cost-scaling relations for the Ostwald process, and from reported costs of plasma reactors. The current estimated cost for the plasma-reactor is 0.90 € W^-1^, based on a recent estimate of Van Rooij *et al.*^105^ for microwave reactors, as well as the cost of power supplies for DBD reactors (about 1.00–2.00 € W^−1^ for a few hundreds of W). The estimated cost for plasma generators is expected to decrease to 0.05 € W^−1^ for large-scale application.^105^

**Fig. 7:**
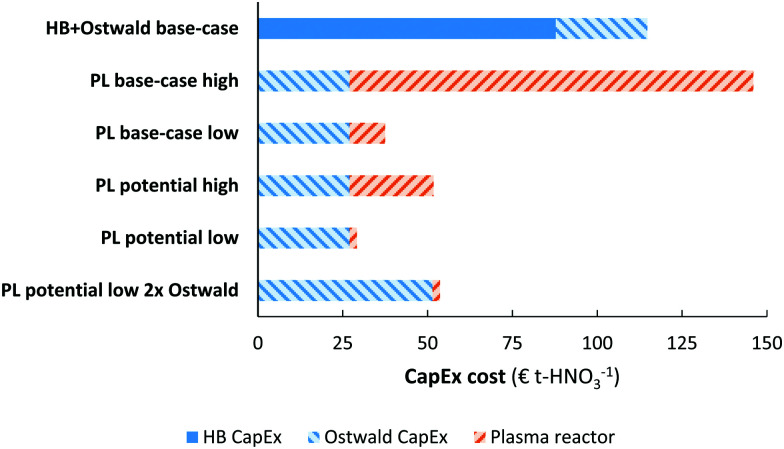
Comparison of the capital expenditure for various HNO_3_ synthesis methods. Cost-scaling numbers from ref. 103 for the electrolysis-based Haber–Bosch process, from ref. 104 for the Ostwald process, and ref. 105 for the plasma reactor. See text for more information. The annuity is assumed to be 10%.

A comparison of the capital expenditure for the electrolysis-based Haber–Bosch process, combined with the Ostwald process, and the plasma-based NO_*X*_ synthesis process is shown in Fig. 7. The ‘high’ case and ‘low’ case refer to a plasma generator cost of 0.90 € W^−1^ and 0.05 € W^−1^, respectively. As shown in Fig. 7, the cost of a PL base-case is nearly on par with the EHB + O base-case. Upon improving the energy consumption from 2.4 MJ mol N^−1^ to 0.5 MJ mol N^−1^ or upon decreasing the cost of the plasma generator, the capital expenditure of the plasma-based process is about half to one third that of the EHB + O base-case. Thus, the plasma-based NO_*X*_ synthesis process has potentially a highly competitive capital expenditure, especially when the cost of the plasma generator becomes as low as 0.05 € W^−1^.

We assumed that the CapEx for the plasma process is similar to that of the Ostwald process (apart from the plasma reactor), due to the similarity in the downstream NO_*X*_ absorption steps. However, the NO_*X*_ concentration may be lower in case of plasma-based NO_*X*_ synthesis (see Fig. 5). Therefore, an additional unit operation may be required to concentrate the produced NO_*X*_ for the plasma-based NO_*X*_ synthesis process. Therefore, we also show the CapEx for the plasma-based NO_*X*_ process (PL) with double the equipment required for downstream NO_*X*_ absorption and conversion to HNO_3_. As shown in Fig. 7, the CapEx of the PL process is lower, even if twice the equipment capacity is required for the NO_*X*_ absorption in the PL process as compared to the EHB + O base-case process.

### Effect of energy consumption

**Fig. 8:**
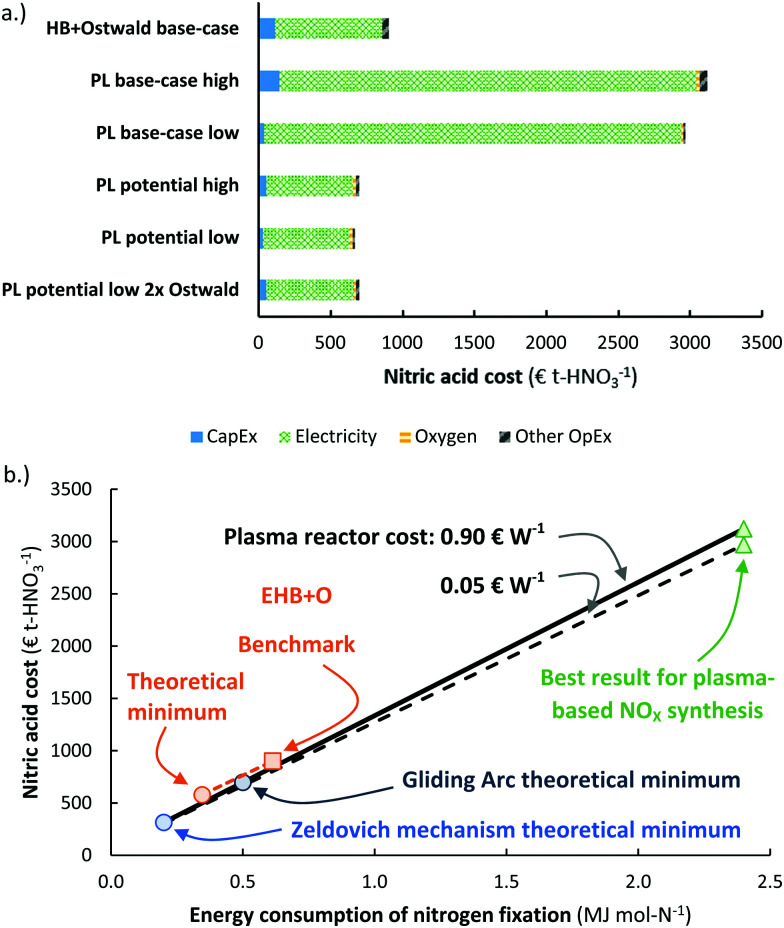
(a) Cost breakdown of the total cost of nitric acid production, for the cases considered in Fig. 7. The ‘high’ case and ‘low’ case refer to a plasma generator cost of 0.90 € W^−1^ and 0.05 € W^−1^, respectively. Process capacity 100 t-HNO_3_ day^−1^, electricity cost 20 € MW h^−1^. Oxygen is added to account for the lower oxygen content in air, as compared to the nitrogen content in air. At the process scale of 100 t-HNO_3_ day^−1^, about 1300 m^3^-O_2_ h^−1^ is required, which costs about 14–28 € t-HNO_3_^−1^.^106^ The operational costs apart from the electricity cost is assumed to be 2% of the CapEx. (b) Effect of the energy consumption of the plasma-based NO_*X*_ synthesis process on the total cost of nitric acid production. The solid and dotted line represent the plasma process with a plasma reactor cost of 0.90 € W^−1^ and 0.05 € W^−1^, respectively. The orange square represents the total cost of nitric acid for a reference electrolysis-based Haber–Bosch process combined with an Ostwald process. Process capacity 100 t-HNO_3_ day^−1^, electricity cost 20 € MW h^−1^.

The energy consumption is another important descriptor for the operational cost of a process (see Fig. 8). The cases presented in Fig. 7 are also shown in Fig. 8a. It is clear that the energy consumption has a major impact on the total cost of HNO_3_ production, and a minor increase in the capital expenditure has little effect on the overall economics on the process. Thus, it is reasonable to focus on the energy consumption of the process.

The effect of the energy consumption on the nitric acid cost in the plasma-based NO_*X*_ synthesis process is shown by the solid and dotted lines in Fig. 8b, from which it follows that the plasma-based NO_*X*_ synthesis process becomes competitive with the electrolysis-based Haber–Bosch process combined with the Ostwald process at an energy consumption of 0.7 MJ mol N^−1^. As listed in Table 1, this is not attainable for thermal plasmas, as these plasmas have a minimum energy consumption of 0.72 MJ mol N^−1^. However, warm plasmas may attain the required energy consumption below 0.7 MJ mol N^−1^ (see Table 1).

### Effect of electricity cost & process capacity

It should be noted that the current market value of HNO_3_ is about 250–350 € t-HNO_3_^−1^, while the predicted cost of HNO_3_ production for the EHB + O base-case and the PL potential low cases is as high as 890 € t-HNO_3_^−1^ and 655 € t-HNO_3_^−1^ for an electricity cost of 20 € MW h^−1^. The relatively low market value of HNO_3_ is mainly due to the low cost of fossil-based feedstocks, such as natural gas and coal.^107^ As shown in Fig. 8b, the CapEx only has a minor effect on the total cost of HNO_3_ production at the process scale considered (100 t-HNO_3_ day^−1^). Thus, the cost of electricity is a common descriptor for sustainable HNO_3_ production from the electrolysis-based Haber–Bosch process combined with the Ostwald process and the plasma-based NO_*X*_ synthesis process, as compared to fossil-based HNO_3_ production.

**Fig. 9:**
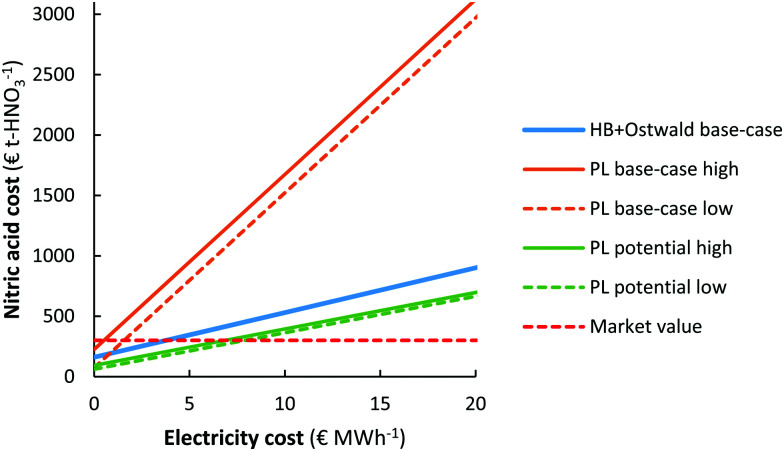
Effect of the electricity cost on the cost of nitric acid production. Process capacity 100 t-HNO_3_ day^−1^. The same cases are considered as in Fig. 7.

The cost of nitric acid production as function of the electricity cost is shown in Fig. 9. It is immediately clear that chemicals produced with electricity require low electricity cost (<5–10 € MW h^−1^) in order to become cost-competitive with fossil-based HNO_3_ production. The lowest solar auction prices in recent years are in the range 15–20 € MW h^−1^, implying the electricity-driven processes may become competitive with fossil-based processes in the upcoming decades.

It should be noted, however, that the cost of HNO_3_ depends on the geographic location. While the market value is as low as 250–350 € t-HNO_3_^−1^ in some locations where the cost of transportation is minimal, the cost at remote locations (*e.g.*, the interior of sub-Saharan Africa) can be multiple times that of the production cost^108,109^ so that electricity driven processes may become favourable at higher electricity cost.

### Effect of process capacity

**Fig. 10:**
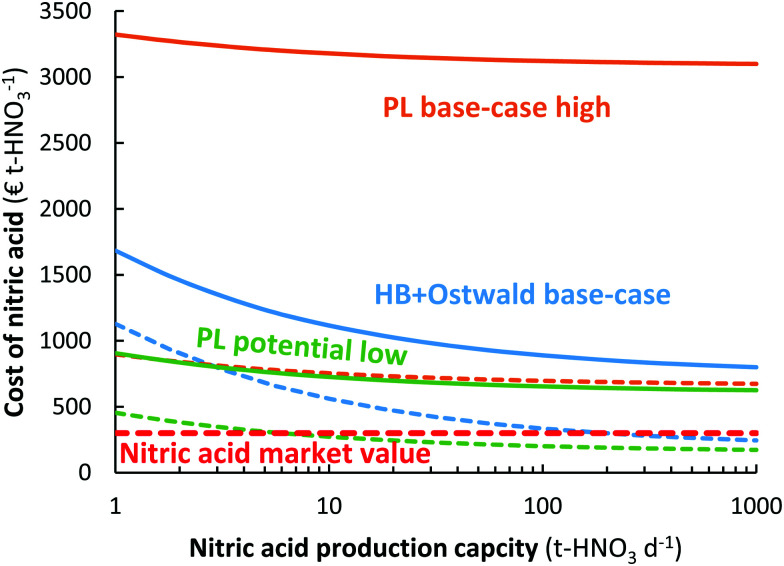
Effect of nitric acid production capacity on the cost of nitric acid for the electrolysis-based Haber–Bosch process combined with the Ostwald process, as well as for the plasma-based NO_*X*_ synthesis process. The full and dotted lines represent an electricity cost of 20 € MW h^−1^ and 5 € MW h^−1^, respectively. The high pressure Haber–Bosch process becomes less energy-efficient upon scale down below 10 t-HNO_3_ day^−1^.^14,110^ The HB + Ostwald base-case, PL base-case, and PL potential case are the same as in Fig. 8.

As shown in Fig. 10, the plasma-based NO_*X*_ synthesis process has the benefit over the Haber–Bosch process combined with the Ostwald process that the capital expenditure for ammonia synthesis is not required. This means there is potential for decentralized HNO_3_ synthesis, instead of importing HNO_3_ to remote locations.^109^ While the Haber–Bosch process suffers from a high CapEx upon scale-down to capacities below 50 t-HNO_3_ day^−1^, the plasma-based NO_*X*_ synthesis process may be scaled down more effectively (see Fig. 10). Hence, plasma-based NO_*X*_ synthesis may be used for decentralized nitrogen fixation. It should be noted, however, that scale-down below 1 t-HNO_3_ day^−1^ also becomes less economical for the plasma-based NO_*X*_ synthesis process, due to an increase in oxygen purification cost upon scale-down.^106^

### Further improving the performance of plasma-based NO_*X*_ synthesis

In recent years, various studies have reported on combination of experimental and modelling work for plasma-based NO_*X*_ synthesis.^33,34,62,91^ This has improved the understanding of the dominant reaction pathways in real plasma reactors under relevant reaction conditions. However, the energy cost of plasma-based NO_*X*_ synthesis remains higher than for the benchmark electrolysis-based Haber–Bosch process combined with the Ostwald process (see Fig. 5). Thus, further performance improvement is required, beyond optimizing experimental conditions, *e.g.* inspired by modelling.

Modelling can, however, also help to improve the reactor design to improve the contacting of gas with plasma so that a larger fraction of gas actually passes through the plasma. This is now often a limitation in for instance gliding arc plasma reactors,^34,99^ thus limiting the overall gas conversion. Such modelling can describe gas flow dynamics, arc plasma behaviour and plasma chemistry, tracing the gas molecules through the reactor. This allows evaluation of the exact plasma conditions to which molecules are exposed, resulting on optimal conversion by the plasma, as recently demonstrated.^33,111^

Besides enhancing the gas fraction passing through the plasma, attention should also be paid to fast quenching, *i.e.* cooling, of the gas downstream of the plasma, avoiding the backward reaction, *i.e.* decomposition of NO_*X*_ to N_2_ and O_2_. The major beneficial effects of fast quenching were recently studied in detail for CO_2_ conversion in plasma,^112^ but the same principle also applies to NO_*X*_ synthesis. In addition, heat integration is required, using the heat released during gas cooling for pre-heating the gas before entering the plasma reactor,^82^.

Finally, as discussed in Section 2.2, catalytic enhancement of plasma-based NO_*X*_ synthesis is an option to increase the NO_*X*_ yield at the same energy input. Such materials should not catalyse the decomposition of NO_*X*_ molecules, as this would even decrease the NO_*X*_ yield as compared to pure plasma-based NO_*X*_ synthesis. Secondly, the use of NO_*X*_ sorbents may be beneficial. Removal of NO_*X*_ species from the plasma environment may prevent the subsequent decomposition of the product by the plasma. Catalyst particles or sorbent particles may be introduced in or after the plasma reactor as a fixed bed, a trickle bed, or a fluidized bed.

## Conclusion

We have evaluated the state-of-the-art for plasma-based NO_*X*_ synthesis. From a techno-economic analysis, it follows that plasma-based NO_*X*_ synthesis is potentially viable for electricity-based HNO_3_ production. As compared to the electrolysis-based Haber–Bosch process combined with the Ostwald process, the plasma-based NO_*X*_ synthesis process benefits from a lower capital expenditure. The current energy cost of ≥2.4 MJ mol N^−1^^91^ is however still too high to be competitive with the electrolysis-based Haber–Bosch process combined with the Ostwald process, which consumes about 0.6 MJ mol N^−1^^15^. Plasma-based NO_*X*_ synthesis will become a highly-competitive alternative to the Haber–Bosch process combined with the Ostwald process, if the energy consumption can be decreased to 0.7 MJ mol^−1^*via* smart reactor design, tuning the chemistry and vibrational kinetics, avoiding back-reactions, or combination with catalysts. Thus, plasma technology may become an effective turnkey technology compatible with intermittent electricity.^113^

## Conflicts of interest

There are no conflicts to declare.
