# Correction: From the Birkeland–Eyde process towards energy-efficient plasma-based NO_*X*_ synthesis: a techno-economic analysis

**DOI:** 10.1039/d3ee90066e

**Published:** 2023-11-27

**Authors:** Kevin H. R. Rouwenhorst, Fatme Jardali, Annemie Bogaerts, Leon Lefferts

**Affiliations:** a Catalytic Processes & Materials, MESA+ Institute for Nanotechnology, University of Twente P.O. Box 217 7500 AE Enschede The Netherlands k.h.r.rouwenhorst@utwente.nl l.lefferts@utwente.nl; b Research Group PLASMANT, Department of Chemistry, University of Antwerp Universiteitsplein 1 B-2610 Wilrijk-Antwerp Belgium fatme.jardali@uantwerpen.be annemie.bogaerts@uantwerpen.be

## Abstract

Correction for ‘From the Birkeland–Eyde process towards energy-efficient plasma-based NO_*X*_ synthesis: a techno-economic analysis’ by Kevin H. R. Rouwenhorst *et al.*, *Energy Environ. Sci.*, 2021, **14**, 2520–2534, https://doi.org/10.1039/D0EE03763J.

There was an error in the conversion factor between ammonia (NH_3_) and nitric acid (HNO_3_), causing the cost of direct plasma-based NO_*X*_ synthesis to be overestimated by a factor of 13.7. The cost per ton of nitric acid was multiplied by 63.01 g per mol-HNO_3_ and divided by 17.031 g per mol-NH_3_, whereas the cost per ton of nitric acid should have been divided by 63.01 g per mol-HNO_3_ and divided by 17.031 g per mol-NH_3_. The subsections ‘Effect of energy consumption’, ‘Effect of electricity cost & process capacity’, and ‘Effect of process capacity’ in the section ‘Comparison of direct plasma-based NO_*X*_ synthesis and the Haber–Bosch process combined with the Ostwald process’ have been rewritten, and Fig. 8–10 have been amended to accommodate the corrections. The overall conclusions of the work have been rewritten as well, as the corrected analysis shows that plasma-based NO_*X*_ synthesis is more much competitive with the Haber–Bosch process combined with the Ostwald process than in our original analysis. References cited herein are as provided in the original article except where new references are indicated.

We are grateful for remarks from Dr B. Heinz (Yara) and Dr Broekhuis (Sabic), making us aware of the mistake.

## Comparison of direct plasma-based NO_*X*_ synthesis and the Haber–Bosch process combined with the Ostwald process

### Effect of energy consumption

The energy consumption is another important descriptor for the operational cost of a process (see Fig. 8). The cases presented in Fig. 7 are also shown in Fig. 8a. It is clear that the energy consumption has a major impact on the total cost of HNO_3_ production, and a minor increase in the capital expenditure has little effect on the overall economics on the process. Thus, it is reasonable to focus on the energy consumption of the process.

**Fig. 8:**
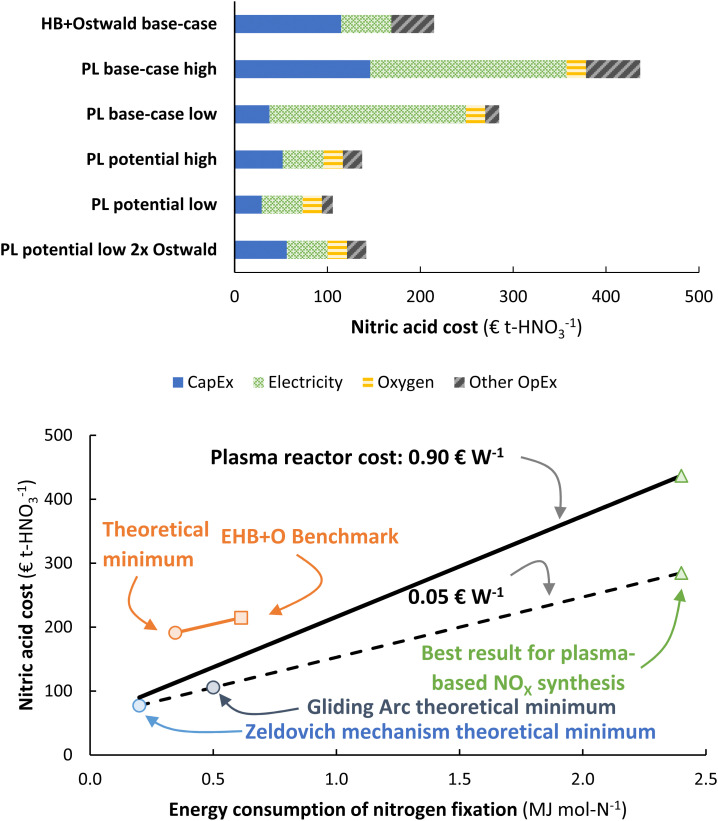
(a) Cost breakdown of the total cost of nitric acid production, for the cases considered in Fig. 7. The ‘high’ case and ‘low’ case refer to a plasma generator cost of 0.90 € W^−1^ and 0.05 € W^−1^, respectively. Process capacity 100 t-HNO_3_ day^−1^, electricity cost 20 € MW h^−1^. Oxygen is added to account for the lower oxygen content in air, as compared to the nitrogen content in air. At the process scale of 100 t-HNO_3_ day^−1^, about 1300 m^3^-O_2_ h^−1^ is required, which costs about 14–28 € t-HNO_3_^−1^.^106^ The operational costs apart from the electricity cost is assumed to be 2% of the CapEx. (b) Effect of the energy consumption of the plasma-based NO_*X*_ synthesis process on the total cost of nitric acid production. The solid and dotted line represent the plasma process with a plasma reactor cost of 0.90 € W^−1^ and 0.05 € W^−1^, respectively. The orange square represents the total cost of nitric acid for a reference electrolysis-based Haber–Bosch process combined with an Ostwald process. Process capacity 100 t-HNO_3_ day^−1^, electricity cost 20 € MW h^−1^.

The effect of the energy consumption on the nitric acid cost in the plasma-based NO_*X*_ synthesis process is shown by the solid and dotted lines in Fig. 8b, from which it follows that the plasma-based NO_*X*_ synthesis process becomes competitive with the electrolysis-based Haber–Bosch process combined with the Ostwald process at an energy consumption of 1.0–1.5 MJ mol N^−1^, depending on the cost of the plasma reactor. Note that a significantly higher energy consumption is acceptable compared to the number (0.7 MJ mol N^−1^) in the paper as published. As listed in Table 1 in the paper as published, this is theoretically attainable for thermal plasmas, which have a minimum energy consumption of 0.72 MJ mol N^−1^. Warm plasmas and non-thermal plasmas may attain an energy consumption even below 0.7 MJ mol N^−1^ (see Table 1).

## Effect of electricity cost & process capacity

The current market value of HNO_3_ is about 250–350 € t-HNO_3_^−1^, while the predicted cost of HNO_3_ production for the EHB + O base-case and the PL potential low cases is as low as 215 € t-HNO_3_^−1^ and 106 € t-HNO_3_^−1^ for an electricity cost of 20 € MW h^−1^, instead of 890 € t-HNO_3_^−1^ and 655 € t-HNO_3_^−1^ in the paper as published. This indicates that the electrolysis-based Haber–Bosch process combined with the Ostwald process and the plasma-based NO_*X*_ process have the potential to be competitive with current market prices from the fossil-based Haber–Bosch process combined with the Ostwald process. The PL base-case has a cost in the range of 285–437 € t-HNO_3_^−1^, implying an improvement in energy efficiency is still required.

The cost of nitric acid production as a function of the electricity cost is shown in Fig. 9. Even at relatively high electricity costs up to 50 € MW h^−1^, the plasma-based NO_*X*_ process can potentially become competitive with market prices. However, electricity prices must be below 10–20 € MW h^−1^ to be competitive with the market value of HNO_3_ considering the relatively high energy consumption in the best results obtained so far for plasma-based NO_*X*_ production. The lowest solar auction prices in recent years are in the range of 15–20 € MW h^−1^ and are expected to decrease further, implying that the electricity-driven processes will become increasingly competitive with current fossil-based HNO_3_ production in the upcoming decades. The lowest potential nitric acid cost *via* the plasma-based NO_*X*_ process is lower than for the electrolysis-based Haber–Bosch process combined with the Ostwald process (see Fig. 9).

**Fig. 9:**
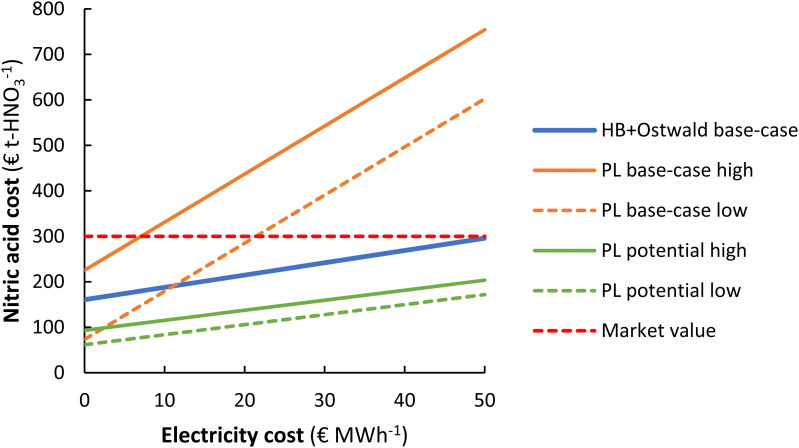
Effect of the electricity cost on the cost of nitric acid production. Process capacity 100 t-HNO_3_ day^−1^. The same cases are considered as in Fig. 7.

It should be noted as discussed in the paper as published, however, that the cost of HNO_3_ depends on the geographic location. While the market value is as low as 250–350 € t-HNO_3_^−1^ in some locations where the cost of transportation is minimal, the cost at remote locations (*e.g.*, the interior of sub-Saharan Africa) can be multiple times that of the production cost^108,109^ so that electricity driven processes may become favorable at higher electricity cost.

## Effect of process capacity

As shown in Fig. 10, the plasma-based NO_*X*_ synthesis process has the benefit over the Haber–Bosch process combined with the Ostwald process that the capital expenditure for ammonia synthesis is not required. This means there is potential for decentralized HNO_3_ synthesis, instead of importing HNO_3_ to remote locations.^109^ While the Haber–Bosch process suffers from a high CapEx upon scale-down to capacities below 50 t-HNO_3_ day^−1^, the plasma-based NO_*X*_ synthesis process may be scaled down more effectively (see Fig. 10). Hence, plasma-based NO_*X*_ synthesis may be used for decentralized nitrogen fixation. It should be noted, however, that scale-down below 1 t-HNO_3_ day^−1^ also becomes less economical for the plasma-based NO_*X*_ synthesis process, due to increasing costs for oxygen purification and the nitric acid absorption column upon scale-down.^1^ Note that this last argument was not discussed in the paper as published.

**Fig. 10:**
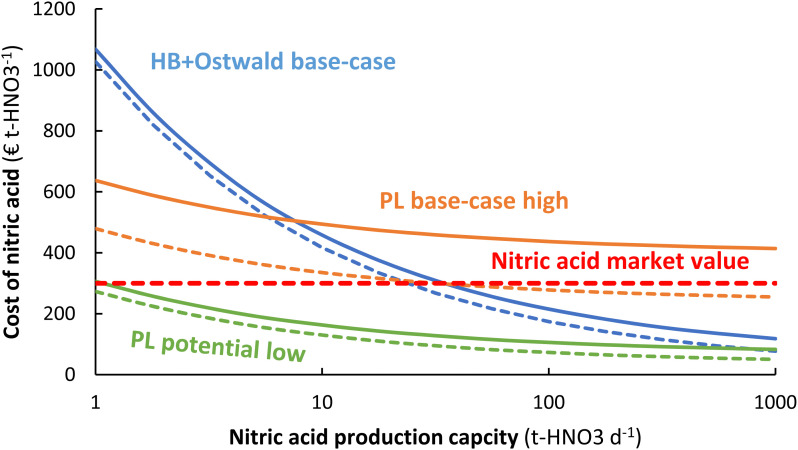
Effect of nitric acid production capacity on the cost of nitric acid for the electrolysis-based Haber–Bosch process combined with the Ostwald process, as well as for the plasma-based NO_*X*_ synthesis process. The full and dotted lines represent an electricity cost of 20 € MW h^−1^ and 5 € MW h^−1^, respectively. The high-pressure Haber–Bosch process becomes less energy-efficient upon scale-down below 10 t-HNO_3_ day^−1^.^14,110^ The HB + Ostwald base-case, PL base-case, and PL potential case are the same as in Fig. 8.

## Conclusions

We have evaluated the state-of-the-art for plasma-based NO_*X*_ synthesis. From a techno-economic analysis, it follows that plasma-based NO_*X*_ synthesis is potentially viable for electricity-based HNO_3_ production. Compared to the electrolysis-based Haber–Bosch process combined with the Ostwald process, the plasma-based NO_*X*_ synthesis process benefits from a lower capital expenditure. The current energy cost of ≥2.4 MJ mol N^−1^ ^91^ in the paper as published is however still too high to be competitive with the electrolysis-based Haber–Bosch process combined with the Ostwald process, which consumes about 0.6 MJ mol N^−1^.^15^ In the meantime, new papers have been published for plasma-based NO_*X*_ synthesis, reporting lower energy consumptions. Kelly *et al.* (new ref. 1 below) report an energy consumption of 2 MJ mol N^−1^ at 3.8% NO_*X*_ outlet concentration in an atmospheric pressure MW (microwave) plasma. Tsonev *et al.* (new ref. 2 below) report an energy consumption of 1.8 MJ mol N^−1^ for a RGA (rotating gliding arc) operating at a pressure of 3 barg at nearly 5% NO_*X*_ outlet concentration. Plasma-based NO_*X*_ synthesis will become a highly competitive alternative to the Haber–Bosch process combined with the Ostwald process, if the energy consumption can be decreased to 1.0–1.5 MJ mol^−1^*via* smart reactor design, tuning the chemistry and vibrational kinetics, avoiding back-reactions, or combination with catalysts. Note that this value was 0.7 MJ mol N^−1^ in the paper as published. Thus, plasma technology may become an effective turnkey technology compatible with intermittent electricity.^113^

The Royal Society of Chemistry apologises for these errors and any consequent inconvenience to authors and readers.

## Supplementary Material
